# Sex Differences on the Pharmacokinetics of Drugs for Children with Chronic Kidney Disease: A Narrative Review

**DOI:** 10.34172/apb.2024.056

**Published:** 2024-06-30

**Authors:** Toktam Faghihi, Farahnak Assadi

**Affiliations:** ^1^Department of Clinical Pharmacy, School of Pharmacy, Tehran University of Medical Sciences, Tehran, Iran, and Pediatrics Center of Excellence, Children’s Medical Center, Tehran University of Medical Sciences, Tehran, Iran.; ^2^Department of Pediatrics, Division of Nephrology, Rush University Medical Center, Chicago, Illinois USA.

**Keywords:** Children, Chronic kidney disease, Pharmacotherapy, Pharmacokinetics, Sex differences

## Abstract

Effective optimal pharmacotherapy requires a comprehensive understanding of the drug’s pharmacokinetic properties. Chronic kidney disease (CKD) influences medication pharmacokinetics. However, whether sex differences exist in the pharmacokinetics of drugs for children with CKD is unknown. The primary aim of this article was to evaluate the effect of sex on pharmacokinetics of drugs commonly used for CKD treatment in children. Secondary outcome was to address the impact of sex in CKD disease progression. Electronic databases, PubMed, EMBASE, Google Scholar, and Web of Science were searched from inception, using Mesh terms in English for sex differences in the pharmacokinetics of drugs in children with CKD. No studies have documented sex-related differences in the pharmacokinetics of drugs for the treatment of CKD in children. As a consequence, it is difficult to predict the effect of sex on pharmacokinetics by extrapolating data from adult studies to children. Evidence to date suggests that girls generally have a higher prevalence and disease progression of CKD when compared to boys regardless of age. Understanding the pharmacokinetics and pharmacodynamics of drugs provides practical consideration for dosing optimal medication regimens. Future kinetic studies are needed evaluating the effect of sex on the pharmacokinetics and pharmacodynamics of drugs in children with CKD.

## Introduction

 It has been illuminated that CKD influences drug pharmacokinetics.^1^ Interestingly, accumulating evidence from epidemiological and clinical studies suggests that sex-related differences exist in the prevalence, course, and progression of chronic kidney disease (CKD) in adults.^2^ In adults, the prevalence, incidence, and disease progression are higher in men than in women.^3^ Among children, girls have been reported to have a higher incidence rate of CKD, defined as estimated glomerular filtration rate (eGFR) < 60 mL/min/1.73 m^2^, than boys because of a faster decline in GFR compared with boys. Certain risk factors for the development and progression of CKD including frequent urinary tract infection (UTI) and autoimmune-related glomerular diseases such as lupus nephritis are also higher in girls than in boys.^4-6^ Similarly, the mortality rate is also substantially higher in girls treated with renal replacement therapy (RRT) compared to boys.^7-9^ Moreover, significant sex differences also exist in the pharmacokinetics and pharmacodynamics of drugs between men and women,^10-13^ but data on sex differences in pediatric pharmacokinetics and drug dosing are lacking.

 Children’s doses cannot be extrapolated directly from adult studies as the pharmacokinetics of many drugs are different in children compared to adults. Pharmacokinetics vary with sex, age, rapid changes in size, body composition, and organ function, particularly during early development.^14,15^ A change in the pharmacokinetics during development can affect drug elimination and exposure predisposing the child to over or under medication leading to severe adverse events or treatment failure.^2,3^

 The lack of pharmacokinetics knowledge complicates finding the correct dosing for the management of children with CKD. This review addresses the influence of sex differences on the pharmacokinetics of drugs and on the incidence of disease progression in children with CKD.

 Electronic databases, PubMed, Embase, and Web of Science have been searched since inception, using Mesh terms English for sex differences in the management and pharmacokinetics of drugs in children and adolescents with CKD. All studies included were published from inception. Of the 70 reviewed articles, 36 met the inclusion criteria and were included in analyses.

###  Sex differences in genetic and epigenetic process

 Studies have documented genetic dispositions for certain diseases associated with CKD in females.^16-18^ Skewed X chromosome inactivation has been shown to predispose females to several autoimmune diseases including lupus nephritis, scleroderma, rheumatoid arthritis, Sjogren’s disease, and myasthenia gravis.^16^ Many genes possess hormone response elements in the region of their target genes, which can interfere with the transcription of target genes. The expression of DNA methylation is also under sex hormones (estrogens and androgens) control.^19^ Hormonal and genetic factors have also been shown to play a relevant role in explaining these differences in CKD, as demonstrated by several experimental studies, showing an overall protective role for estrogens and progesterone.^20-22^

 Environmental exposures to toxins, air pollution, chemical, microbial, allergens, and unhealthy dietary habits can increase the risk of developing CKD through DNA methylation, suggesting this environmental interaction or epigenetic dysregulation may play an important role in the sex-related differences in CKD amongst children.^18,19^

 Current screening standards including measurement of eGFR and urinary biomarkers such as beta-2 microglobulin and kidney injury molecule (KIM-1) are widely used for CDK detection, but it is difficult to predict CKD precisely.^23^ Evidence-based medicine requires gender heterogeneity to be taken into account in CKD deterioration to inform the risk assessment, monitoring, and prognosis.

###  Sex differences in susceptibility to CKD 

 The majority of glomerular diseases show a male bias, except lupus nephritis, which is strongly female-predominant.^24^ Studies have shown that collagen-vascular diseases and autoimmune disorders affect females three to ten times more than males.^25^ Environmental stressors-induced epigenetic alteration such as chemical exposures, drugs, and infections during pregnancy, ureter-placental hypoxia, and maternal under-nutrition may lead to prematurity and low birth weight infants and the risk of long-term hypertension and metabolic syndrome leading to the development of CKD, in children and adults.^18,19,26,27^

 UTI is also strongly sex-biased, with girls being 20 to 40 times to have a UTI than boys of the same age.^5,6,28^ Being female significantly influences immune response to diseases at mucosal surfaces. Sex hormones, sex differences, sex chromosomes, and sexual dimorphism all contribute to the development of CKD.^16,17^

 Sex differences also exist in susceptibility to metabolic syndrome with female children being at a higher risk of developing obesity, dyslipidemia, type 2 diabetes, and CKD.^29-31^ Variable epigenetic background, diet, levels of physical activity, and levels of estrogens may influence the higher prevalence of metabolic syndrome in females than in men.^32^

###  Sex differences in renal physiology

 Clinical studies have demonstrated sex differences in kidney size morphology and hemodynamic functions. Total renal mass and the renal cortex and proximal tubules are larger in males than in females.^20^ The contribution of these structural differences to sex-related variation in renal physiology may also account for a higher incidence rate of CKD progression in female children.

###  Sex differences in the pharmacokinetics of drugs frequently used to treat CKD in children

 In contrast to adults, there is no information in the literature about sex-related differences in the pharmacokinetics of drugs for the treatment of CKD in children. As a consequence, it is difficult to predict the impact of sex on pharmacokinetics by extrapolating data from adult studies in children.

###  Influence of CKD on medication pharmacokinetics

 CKD influences multiple pharmacokinetic parameters, which needs to be considered for commonly used medications in this population.^1^ Understanding the pharmacokinetics (PK) properties and the study of the absorption, distribution, metabolism, and excretion (ADME) processes of a drug is essential for effective optimal pharmacotherapy in patients with CKD.

 The pharmacokinetics of many drugs in children is different compared to adults.^12^ Children have a lower body weight and organ size and faster growth and development during early development, which can significantly affect drug absorption, distribution, and elimination.^12^

**Figure 1:**
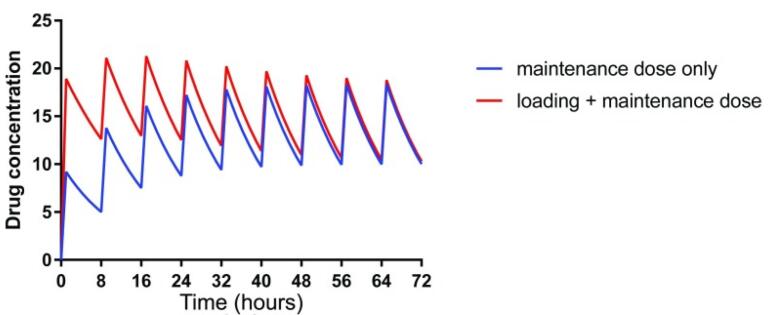
Loading dose decreases the time to achieve target concentration (Used with permission, Robert et al.^7^

**Figure 2:**
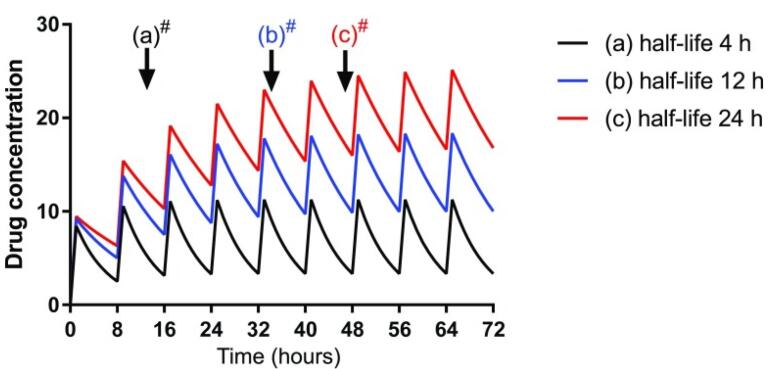
An increase in a drug’s t_1/2_ prolongs the time to achieve steady-state plasma concentrations with maintenance dosing (Used with permission, Robert et al.^7^

 In general, the renal drug clearance depends on GFR, tubular reabsorption capacity, and tubular excretion. In CKD, in the absence of pharmacokinetics properties, drugs that are primarily excreted by GFR such as aminoglycosides, dose adjustment can be made by either decreasing the initial dose or increasing the dosing interval. However, in some patients with active infection, a higher initial loading dose is required to rapidly achieve therapeutic concentration.^33^ A loading dose decreases the time to achieve the target concentration. The plasma drugs’ half-time (t_1/2_) is often prolonged in adult patients with CKD because of reduced GFR. Increasing the t_1/2_ delays the time to achieve steady-state plasma concentrations and results in higher plasma concentrations. Loading dose decreases the time to achieve the target therapeutic plasma concentration (Figure 1). An increase in a drug’s t_1/2_ prolongs the time to achieve steady-state plasma concentrations with maintenance dosing. Failure to reduce the maintenance dose or frequency in CKD patients with the longer t ½ may predispose them to adverse drug reactions (Figure 2).

 The volume of distribution (V_d_) and clearance (CL) are the two most important parameters of the pharmacokinetics of drugs. Both Vd and CL also changes in patients with CKD.^1^

 In general, drug distribution is dependent on the extent of protein binding, lipophilicity or water solubility, renal blood flow, membrane permeability, and tissue uptake.^9-12,16^ Increased protein binding decreases the free concentration and fraction of drugs, thereby limiting the capacity of the active drug to diffuse more easily across the cell membranes. In CKD, uremic toxins displace some drugs from protein binding sites leading to increased unbound fraction. Likewise, phenytoin does not need dose adjustment in patients with reduced GFR, however free drug concentration should be monitored instead of total drug concentration in CKD patients.^34^

Table 1 summarizes the pharmacokinetic parameters and the impact of sex on pharmacokinetics of commonly administered drugs in adults with stage 1-4 CKD.^35-58^

**Table 1:** Sex-related differences in the pharmacokinetics of frequently prescribed drugs in patients with CKD

**Drug class**	**BA (%)**	**PB (%)**	**Vd (L/kg)**	**t**_1/2_** (h)**	**Adults**	**Children**
Antihypertensives						
Captopril	40-90	25-30	0.7	6-12	No sex-related effect^35^	Unknown
Enalapril	40	50	21	42-55	Women may have higher oral bioavailability, lower clearance, and smaller *Vd*^36^	Unknown
Metoprolol	43-69	10-12	3.2-5.6	2-3	Faster clearance in women than in men^37^	Unknown
Propranolol	25	90	4	6-8	No sex-related effect^38^	Unknown
Nifedipine	56-77	92-98	0.6-0.8	2-3	No sex-related effect^39^	Unknown
Amlodipine	65-90	85-90	20-22	30-50	No sex-related effect^40^	Unknown
Prazocin	43-82	92-96	0.5	2-3	No sex-related effect^41^	Unknown
Labetolol	20-30	50	3.3-7.9	5-6	No sex-related effect^42^	Unknown
Diuretics						
Furosemide	50-70	95-99	0.2-0.5	> 8	No sex-related effect^43^	Unknown
Chlorothiazide	Poor	40-68	4-13	40-60	No sex-related effect^44^	Unknown
Spironolactone	80-90	> 90	5-7	2-26	No sex-related effect^45^	Unknown
Digoxin	70-80	20-30	2.5-7.5	30-120	Lower clearance and smaller *Vd*in women^46^	Unknown
Antibiotics						
Cefotaxime	-	32-44	0.2-0.4	1.9-5.8	No sex-related effect^47^	Unknown
Ciprofloxacin	60-66	20-40	2-3	4.2-5.1	Lower clearance and longer t ½ in females^48^	Unknown
Erythromycin	30-65	80-90	0.64	5-6	Lower bioavailability, larger *Vd*, and longer t_1/2_ in females^49^	Unknown
Quinolones	70-90	20-40	1.8-2.1	13-17	Lower *Vd*and longer* t½* in females during^50^ pregnancy	Unknown
Vancomycin	< 10	40-60	0.7-0.9	28-36	No sex-related effect^51^	Unknown
Anticonvulsants						
Phenobarbital	95-100	20-45	0.63	> 120	No sex-related effect^52^	Unknown
Phenytoin	40-60	87.8-91.9	0.4-0.6	0.5-0.8	No sex-related effect^53^	Unknown
Anticoagulants						
Heparin	-				Lower clearance and *Vd*in women^54^	Unknown
Warfarin	-	99	0.14		Lower clearance and smaller*Vd* in women^55^	Unknown
Immunosuppressives						
Cyclosporine	5-89	96-99	4-6	6.2-15.8	Lower clearance in women, suggesting higher rate of nephrotoxicity compared to men^56^	Unknown
Methylprednisolone	81-110	40-60	24	2.6-4.3	Clearance is higher and as a consequence, half-life is lower in women, suggesting higher rate of nephrotoxicity compared to men^57^	Unknown
Mycophenolate	75-87	82	3.6-4	14-18	Slower clearance and highert ½ in females^58^	Unknown

Pharmacokinetic data presents adult data. CKD, chronic kidney disease; BA, bioavailability for oral formulations; PB, protein binding; v_d_, volume of distribution; t_1/2_, half-life; CCB, calcium channel blockers

###  Relevance to patient care and clinical practice

 Impaired renal function can significantly alter the pharmacokinetics and pharmacodynamics of drugs, putting patients at risk for drug toxicity or treatment failure if appropriate dosing adjustments are not applied. The incidence rate and progression of CKD is higher in girls than in boys worldwide. There is no information in the literature about sex-related differences in the pharmacokinetics of drugs for the treatment of CKD in children.

 Understanding the pharmacokinetics and pharmacodynamics of drugs provides practical consideration for dosing optimal medication regimens.

## Discussion

 The incidence rate and progression of CKDis higher in girls than in boys worldwide. There is no information in the literature about sex-related differences in the pharmacokinetics of drugs for the treatment of CKD in children.

## Conclusion

 The prevalence of CKD is higher in girls than boys but reverses when they reach adulthood, possibly owing to the protective effects of estrogens. More girls than boys start RRT because of faster CKD progression and because they are more likely to experience frequent UTIs. Girls are at greater risk of CKD progression and CVD disease.

 In contrast to adults, there is no information in the literature about sex-related differences in the pharmacokinetics of drugs for the treatment of CKD in children.

 The lack of pharmacokinetic studies in children with CKD makes it very difficult to predict the optimum therapeutic dosing. Prescribing drug doses extrapolated from adult studies increases the risk of adverse events due to overmedication or lead to treatment failure due to sub-therapeutic exposure.

 Future kinetic studies are eagerly needed to anticipate better and model drug-response relationships in boys and girls.

## Competing Interests

 Theauthors have no relevant financial or non-financial interests to disclose.

## Ethical Approval

 Not applicable.
